# Efficacy and safety of stem cell therapy for dry eye disease: a systematic review and meta-analysis

**DOI:** 10.1186/s13287-026-04915-8

**Published:** 2026-05-01

**Authors:** Kai-Yang Chen, Hoi-Chun Chan, Chi-Ming Chan

**Affiliations:** 1 Department of General Medicine, Chang Gung Memorial Hospital (Linkou Branch), Taoyuan, Taiwan; 2 School of Pharmacy, China Medical University, Taichung, Taiwan; 3 Department of Ophthalmology, Cardinal Tien Hospital, New Taipei City, Taiwan; 4 School of Medicine, Fu Jen Catholic University, New Taipei City, Taiwan

**Keywords:** Dry eye disease (DED), Stem cell therapy, Mesenchymal stem cells (MSCs), Exosomes, Tear production, Ocular surface inflammation

## Abstract

**Introduction:**

Dry eye disease (DED) is a multifactorial ocular surface disorder characterized by loss of tear film homeostasis, inflammation, neurosensory abnormalities, and epithelial damage. Despite the availability of topical immunomodulators and procedural interventions, a substantial proportion of patients with moderate-to-severe or refractory DED experience persistent symptoms and inadequate ocular surface recovery. Stem cell–based therapies, particularly mesenchymal stem cells (MSCs) and MSC-derived exosomes, have emerged as regenerative and immunomodulatory strategies aimed at restoring epithelial integrity and tear film stability rather than providing solely symptomatic relief. We conducted a systematic review and meta-analysis to evaluate the clinical efficacy and safety of stem cell and stem cell–derived therapies in human DED.

**Methods:**

This study followed PRISMA 2020 guidelines and was prospectively registered in PROSPERO (CRD420251057372). Six databases were searched from inception to May 14, 2025. Eligible studies were peer-reviewed human clinical investigations evaluating stem cell–based interventions for DED and reporting objective efficacy outcomes such as Schirmer test, tear break-up time (TBUT), corneal fluorescein staining (CFS), or patient-reported outcomes including the Ocular Surface Disease Index (OSDI). Pooled mean differences (MDs) or standardized mean differences (SMDs) with 95% confidence intervals (CIs) were calculated. Statistical heterogeneity was assessed using the I² statistic. Risk of bias was evaluated using RoB 2 for randomized controlled trials and ROBINS-I for non-randomized studies.

**Results:**

Six studies comprising 131 patients were included. Stem cell–based therapies demonstrated significant improvements in tear production, tear film stability, epithelial integrity, and symptom burden. Schirmer test improved by MD = 4.70 mm (95% CI, 4.18–5.22; *p* < 0.001; I² = 12.59%), indicating a consistent enhancement of aqueous tear secretion. TBUT showed a large standardized improvement with pooled SMD = 1.125 (95% CI, 0.821–1.428; p < 0.001), although randomized trials demonstrated smaller effect sizes than non-randomized studies. OSDI scores decreased by MD = −11.44 points (95% CI, −22.71 to −0.17; *p* = 0.047), reflecting symptomatic improvement but with substantial between-study variability. Corneal fluorescein staining decreased by MD = −1.04 (95% CI, −1.23 to −0.84; *p* < 0.001; I² = 0%), supporting epithelial recovery. No serious treatment-related adverse events were reported; however, safety reporting was heterogeneous and follow-up durations were limited.

**Conclusion:**

Stem cell and stem cell–derived therapies are associated with significant improvements in both objective and subjective outcomes in DED and demonstrate a favorable short-term safety profile. Nevertheless, heterogeneity in cell source, delivery route, dosage, and study design limits generalizability. Larger, rigorously designed randomized trials with standardized protocols and longer follow-up are required to confirm efficacy and establish long-term safety.

**Supplementary Information:**

The online version contains supplementary material available at 10.1186/s13287-026-04915-8.

## Introduction

The etiology of dry eye disease (DED) – characterized by loss of tear film homeostasis and ocular symptoms – includes neurosensory abnormalities, ocular surface inflammation and damage, and tear film instability and hyperosmolarity [1]. Because sex hormones influence lacrimal gland function, meibomian gland physiology, and ocular surface immune homeostasis, DED is generally more prevalent in females than in males. Population-based estimates vary substantially by case definition and setting, but adult prevalence is commonly reported in the range of approximately 12% to 22%, with increasing prevalence in older age groups [2, 3]. Dry eye disease is increasingly recognized as a systemically linked, multifactorial disorder with important metabolic and vascular comorbidities. Our previous meta-analysis also demonstrated a significant association between DED and diabetes mellitus, reinforcing the concept that ocular surface dysfunction frequently co-exists with systemic disease and may share common pathogenic pathways [4]. Over the past decade, a wide spectrum of pharmacological and procedural interventions has been introduced for DED management, including topical immunomodulators, punctal occlusion, thermal pulsation systems, LipiFlow, intense pulsed light, botulinum toxin A, and peri-cataract dry eye optimization. Our group has conducted multiple systematic reviews and meta-analyses across these modalities, consistently showing that although many interventions improve signs and symptoms, a substantial proportion of patients—particularly those with moderate-to-severe or refractory disease—continue to experience persistent ocular surface damage and inadequate symptom control [5–9]. Despite this wide range of treatments, these approaches may be insufficient for severe or treatment-resistant cases, where regenerative therapies such as mesenchymal stem cell (MSC)-based approaches offer potential for enhanced ocular surface repair and inflammation control [10].

To reduce inflammation and the immunological response, topical medications such as cyclosporine and glucocorticoids are often the mainstay of medical therapy for dry eye. However, dry eye is a chronic, long-term condition. The symptoms of the ocular surface may be temporarily alleviated by these therapies, but they cannot be completely eliminated. In parallel with advances in pharmacotherapy, regenerative and advanced therapies have emerged as transformative strategies in ophthalmology. Our previous work has summarized the role of stem cell–based approaches across a broad range of ocular diseases, as well as gene therapy, retinal prostheses, immune checkpoint modulation, and other cutting-edge modalities, highlighting their potential to shift management from purely palliative to mechanistically restorative paradigms [11–14].

Stem cell–based therapies offer a restorative approach to DED by modulating ocular-surface inflammation, promoting epithelial repair, and helping to reestablish tear film homeostasis [15]. In this context, MSCs exert their effects predominantly through paracrine and immunomodulatory mechanisms rather than long-term engraftment or broad tissue regeneration, making them particularly relevant for DED, where chronic inflammation and epithelial dysfunction are key drivers of disease [16]. MSCs can be isolated from several tissue sources, such as bone marrow, adipose tissue, and umbilical cord, which provide clinically accessible cell populations for ocular applications [17]. However, cell-based MSC therapies still carry inherent risks, including immune rejection, microvascular obstruction, and theoretical concerns about ectopic tissue formation [18]. MSC-derived exosomes have emerged as a promising cell-free alternative that preserves the trophic and immunomodulatory properties of their parent cells while minimizing risks related to uncontrolled proliferation or embolic events [19]. These nanosized vesicles can deliver bioactive proteins, lipids, and nucleic acids to ocular surface tissues, offering a potentially safer and more standardized regenerative strategy for DED [20].

Despite growing interest in regenerative strategies for treating DED. To our knowledge, no prior published systematic review and meta-analysis has specifically synthesized the clinicalhuman evidence on stem cell and stem cell-derived therapies for DED. The existing literature is largely composed of preclinical studies, small-scale clinical trials, and narrative reviews, lacking an integrated synthesis of evidence across species and study designs. This gap highlights the need for a structured analysis to inform clinical translation and future research. Therefore, the aim of this systematic review and meta-analysis is to evaluate the efficacy and safety of stem cell and stem cell-derived therapies for DED.

## Methodology

### Study design

This study was conducted as a systematic review and meta-analysis to evaluate the safety and efficacy of stem cell therapy in the treatment of DED. The review was performed in accordance with the Preferred Reporting Items for Systematic Reviews and Meta-Analyses (PRISMA) guidelines. This systematic review and meta-analysis was conducted in accordance with the Preferred Reporting Items for Systematic Reviews and Meta-Analyses (PRISMA) 2020 statement and was prospectively registered in PROSPERO (CRD420251057372).

### Search strategy

A comprehensive literature search was conducted in six databases – PubMed, Google Scholar, Embase, Web of Science, Scopus, and the Cochrane Library – for studies published from inception to May 14, 2025. Search terms included combinations of the following keywords and MeSH terms: “dry eye disease,” “keratoconjunctivitis sicca,” “aqueous-deficient dry eye,” “stem cell therapy,” “mesenchymal stem cells,” “MSC,” “adipose-derived stem cells,” “Sjogren syndrome” and “regenerative therapy” (Supplementary table S1). Boolean operators AND/OR were used to combine terms. Additionally, references of included articles were manually screened for relevant studies.

### Eligibility criteria

Studies were included only if they met the following inclusion criteria:

Original peer-reviewed studies published in English.

Studies evaluating stem cell-based interventions (including stem cell-derived therapies) for the treatment of DED.

Studies reporting clinical or histological efficacy outcomes, such as Schirmer tear test (STT), tear break-up time (TBUT), ocular surface disease index (OSDI) score, or corneal fluorescein staining (CFS).

Studies reporting safety outcomes and adverse events.

Studies involving human participants with DED, including clinical trials and prospective interventional studies.

Any study that did not meet one or more of the above conditions was eliminated. The same approach was applied to the exclusion criteria: studies were exempted from inclusion if they met any of the following conditions:

Review articles, case reports, editorials, or conference abstracts.

Studies that did not evaluate either stem cell therapy or stem cell-derived therapy.

Studies that did not provide data for DED alone but generalized the symptoms of both oral and eye dryness.

Studies without clear outcome measures or safety reporting.

Studies where stem cell therapy was combined with other experimental therapies, making individual assessment unfeasible.

### Data extraction

Two independent reviewers (K.Y.C and H.C.C.) extracted the following data from each eligible study using a standardized form: author name, year of publication, study design, sample size, stem cell type and delivery method, safety findings, and efficacy outcomes (e.g., STT, TBUT, OSDI and CFS). Disagreements were resolved by consensus with the third author (C.M.C.).

### Quality assessment

Quality assessment of the included studies was conducted using the RoB 2 tool for randomized controlled trials and ROBINS-I for non-randomized studies. RoB 2 tool assesses five domains for each study: (1) randomization process, (2) deviations from intended interventions, (3) missing outcome data, (4) selection of the reported results, and (5) measurement of the outcome. Conversely, the seven domains examined by ROBINS-I are: (1) confounding, (2) selection of participants, (3) classification of interventions, (4) deviations from intended interventions, (5) missing data, (6) measurement of outcomes, and (7) selection of the reported results.

### Data synthesis and statistical analysis

Due to heterogeneity in study populations, stem cell sources, administration routes, and outcome measures, both qualitative synthesis and quantitative meta-analysis were performed where appropriate. For the meta-analysis, when possible, pooled analyses of continuous outcomes such as STT scores, TBUT, CFS and OSDI were performed using Comprehensive Meta-analysis software v3.7. Mean differences (MD) with 95% confidence intervals (CI) were calculated. For continuous outcomes, we prioritized between group effect estimates for randomized controlled trials. When trials reported baseline and follow-up values for both intervention and control groups, we calculated the between group difference in change from baseline. When only post intervention values were reported, we used the between group mean difference at follow-up. For single arm prospective studies without a comparator, we calculated within group change from baseline as a separate synthesis and did not pool single arm pre post effects together with randomized between group estimates unless a consistent estimand could be reconstructed across designs. A random-effects model was primarily used due to expected heterogeneity in study design and interventions, with fixed-effect models applied only for sensitivity analysis where appropriate. For each outcome, the overall pooled effect size was calculated using only the final follow-up time point reported in each study to ensure statistical independence and to avoid multiple contributions from the same cohort. Time-specific subgroup analyses were performed separately where relevant. Statistical heterogeneity was assessed using the I² statistic, with values above 50% indicating substantial heterogeneity. A two-tailed significance level of *p* < 0.05 was considered statistically significant for all pooled estimates. Because fewer than 10 studies were available for each outcome, formal statistical tests for funnel-plot asymmetry were considered underpowered and were not interpreted as reliable indicators of publication bias.

## Results

### Search outcomes

**Fig. 1:**
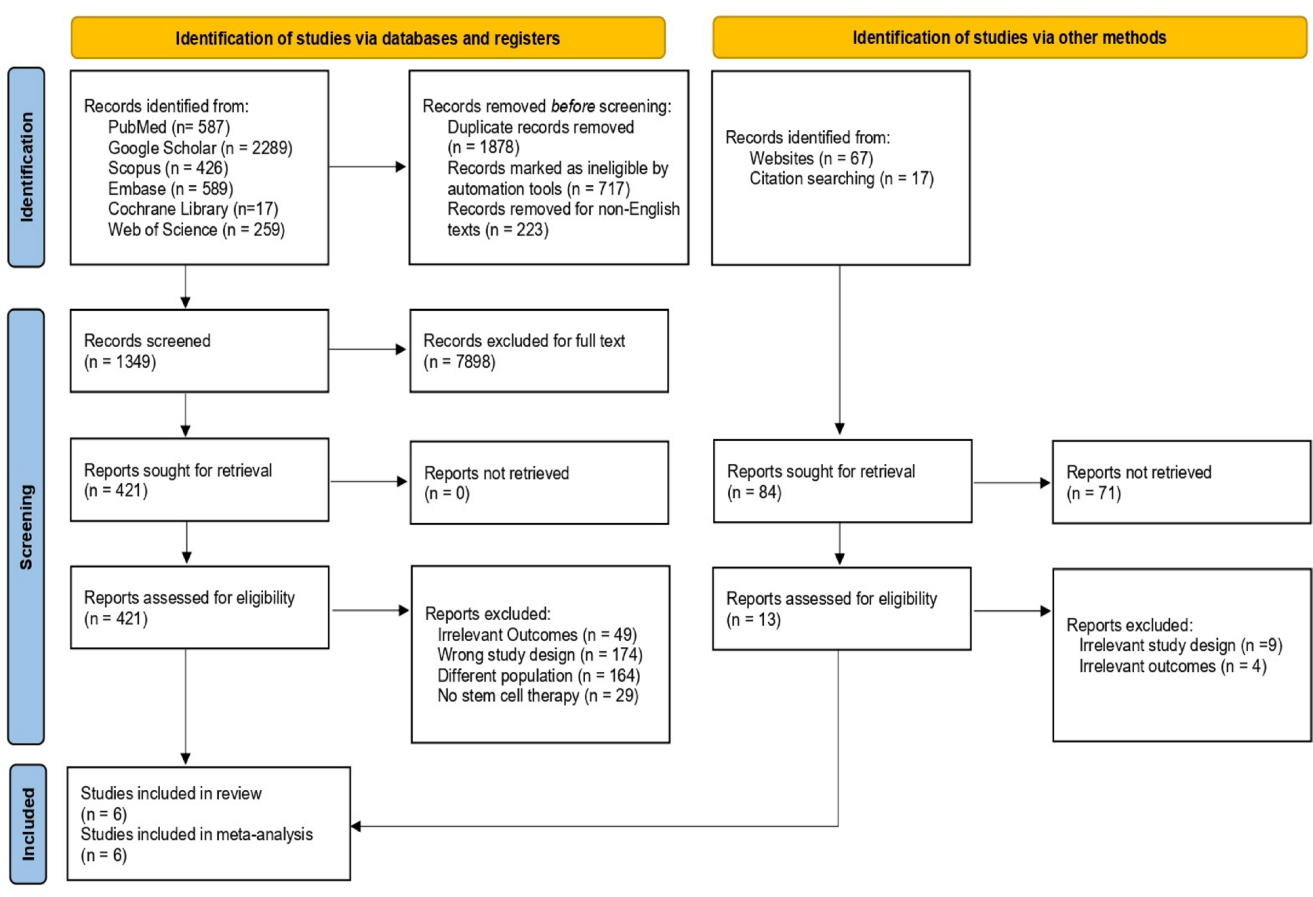
PRISMA flow chart

The PRISMA flowchart below illustrates the study selection process for this review. A total of 4,167 records were identified through database and register searches, with 1,878 duplicates removed and 717 entries automatically excluded. Similarly, 223 articles were eliminated as they were published in languages other than English. Of the remaining 1,349 records, 928 were excluded after title and abstract screening as they were deemed irrelevant to our study objective. The remaining 421 full-text reports were sought for retrieval. All were retrieved and assessed based on the predefined eligibility criteria. The process led to exclusion of 415 articles as follows: assessed other interventions other than stem cell therapy or stem cell-derived therapy (29), assessed patients without DED (164), reported irrelevant outcomes (49), and were secondary studies without primary data (174). Additionally, 84 records were identified via other sources, but 71 could not be retrieved as they were blog posts. Of the 13 assessed, 9 were excluded due to study design and 4 due to irrelevant outcomes. Ultimately, a total of 6 studies were included in the final synthesis (Fig. 1).

### Characteristics of the included studies

Six studies involving 131 patients were ultimately included in this review [21–26]. These studies utilized a wide range of stem-cell-based therapeutic products that varied by tissue source and cell type. Assessed stem-cell-based therapeutic products were adipose-derived MSCs, umbilical cord-derived MSCs, bone marrow-derived MSCs, corneal epithelial stem-cell-derived products, and Wharton’s Jelly-derived MSC exosomes. These therapeutic products ranged from live MSCs (administered systemically, topically, and locally) to acellular derivatives such as exosomes and supernatants. In particular, adipose-derived MSCs were utilized in lacrimal gland injection studies, umbilical cord–derived MSCs and Wharton’s Jelly MSC exosomes were used in topical applications, bone marrow MSCs were delivered systemically, and cadaver-derived corneal epithelial stem cells were used to produce an acellular supernatant applied topically. Table 1 summarizes the characteristics of the included studies.

**Table 1:** Characteristics of the included studies.

Author	Year	Study Design	Population Type and Number	Intervention	Safety Outcomes	Efficacy Outcomes	Results with Stats	Conclusion
Møller-Hansen et al. [22]	2021	Open-label clinical trial	7 patients with aqueous deficient dry eye disease (ADDE)	Single transconjunctival injection of allogeneic ASCs (22 million cells/ml; max 50% of LG volume) into one lacrimal gland	No adverse events related to the treatment were observed during a 126-day follow-up	OSDI, Tear osmolarity, TBUT, Schirmer’s I test, Oxford grading scheme	Significant decrease in OSDI from 58.9 ± 20.6 to 34.1 ± 21.6 (*p* < 0.002); Tear osmolarity decreased from 312.9 ± 10.4 to 291.6 ± 10.9 mOsm/L (*p* < 0.002); TBUT increased from 3.7 ± 1.5 s to 7.1 ± 1.9 s (*p* < 0.002); Schirmer’s I test improved from 4.6 ± 0.7 to 8.1 ± 3.1 mm/5 min (*p* < 0.03); Oxford grading scheme showed a non-significant trend from 2.4 ± 0.7 to 1.3 ± 1 (*p* < 0.10)	Injection of allogeneic ASCs into the LG is safe and showed promising results in improving both subjective symptoms and objective signs of ADDE. Further randomized trials are warranted.
Weng et al. [24]	2012	Human prospective clinical study	22 patients with refractory dry eye due to chronic GVHD	IV infusion of MSCs	No serious adverse events reported	OSDI score, STT, clinical symptoms, peripheral blood CD8+ CD28− T-cell and Th1/Th2 cytokine profiling	- 12/22 patients (54.55%) showed clinical improvement - OSDI scores and STT improved significantly in responders - Increased CD8 + CD28 − T cells in peripheral blood of responders - Responders showed elevated Th1 cytokines (IL-2, IFN-γ), reduced Th2 cytokines (IL-4, IL-10) - In vitro, MSCs induced differentiation of CD8 + T cells into CD8 + CD28 − cells	MSC therapy improved dry eye symptoms in over half of cGVHD patients, likely via immunomodulation and CD8 + T cell reprogramming. Promising systemic cell therapy for immune-mediated DES.
Møller-Hansen[23]	2024	Double-blinded randomized clinical trial with observation arm	Severe SS-related DED; ASCs (*n* = 20), vehicle (*n* = 20), observation (*n* = 14)	Single LG injection of ASCs or vehicle (Cryostor^®^ CS10)	No serious adverse events reported	OSDI, NIBUT, TMH, Schirmer’s test, Oxford grading scheme, tear osmolarity	Significant OSDI ↓ vs. observation; ↑ NIBUT at 4 weeks and 12 months; improved Schirmer and staining vs. baseline (*p* < 0.05)	ASC injections improve both subjective and objective DED signs compared with observation; mechanism of action requires further investigation
Li [37]	2023	Randomized, triple-blind, placebo-controlled clinical trial	Primary SS patients; ADSCs (*n* = 35), placebo (*n* = 39); total *n* = 74 (64 completed)	Local ADSC injection into bilateral glands at 3 time points	No severe adverse events were reported; treatment was well tolerated	ESSDAI, ESSPRI, gland secretion, IgG/IgM, C3/C4, ESR	Significant ESSDAI and ESSPRI improvements; gland secretion ↑ at 3 months; significant ↓ in IgG, IgM, ESR (*p* < 0.05)	ADSCs provide short-term relief of dryness and improve systemic and subjective symptoms in pSS
Habibi [26]	2025	Phase 1 & 2, triple-blinded, randomized trial	Primary SS-related DES; 8 eyes (treated), 8 eyes (control)	Topical MSC-derived exosome drops, 10 µg, BID for 2 weeks	No adverse safety findings were reported; safety was assessed via ophthalmic examinations	OSDI, Schirmer, TBUT, fluorescein score, cytokine levels	Significant ↑ in EGF, ↓ IL-6, MMP-9; improved clinical scores (*p* < 0.05)	Safe and effective for SS-DES; improves symptoms and reduces inflammation
Zhang [25]	2025	Open-label, prospective, single-arm, self-controlled trial	NSDE (*n* = 11) and SSDE (*n* = 5) patients	Umbilical cord MSC eye drops, BID for 2 weeks	No serious AEs reported	OSDI, TMH, NIBUT, SIT, CFS, lipid layer, gland function	Significant improvements in most primary and secondary endpoints (*p* < 0.05)	MSC eye drops show promising therapeutic potential for refractory DED

DED: Dry eye disease, MSC: Mesenchymal stem cells, TBUT: Tear break-up time, OSDI: Ocular surface disease index, CFS: Corneal fluorescein staining, STT: Schirmer tear test, NIBUT: Non-invasive tear break-up time, TMH: Tear meniscus height, ESSDAI: EULAR Sjögren’s Syndrome Disease Activity Index, ESSPRI: EULAR Sjögren’s Syndrome Patient Reported Index, ESR: Erythrocyte sedimentation rate, IL-6: Interleukin-6, MMP-9: Matrix metalloproteinase-9

### Quality appraisal of studies

**Fig. 2:**
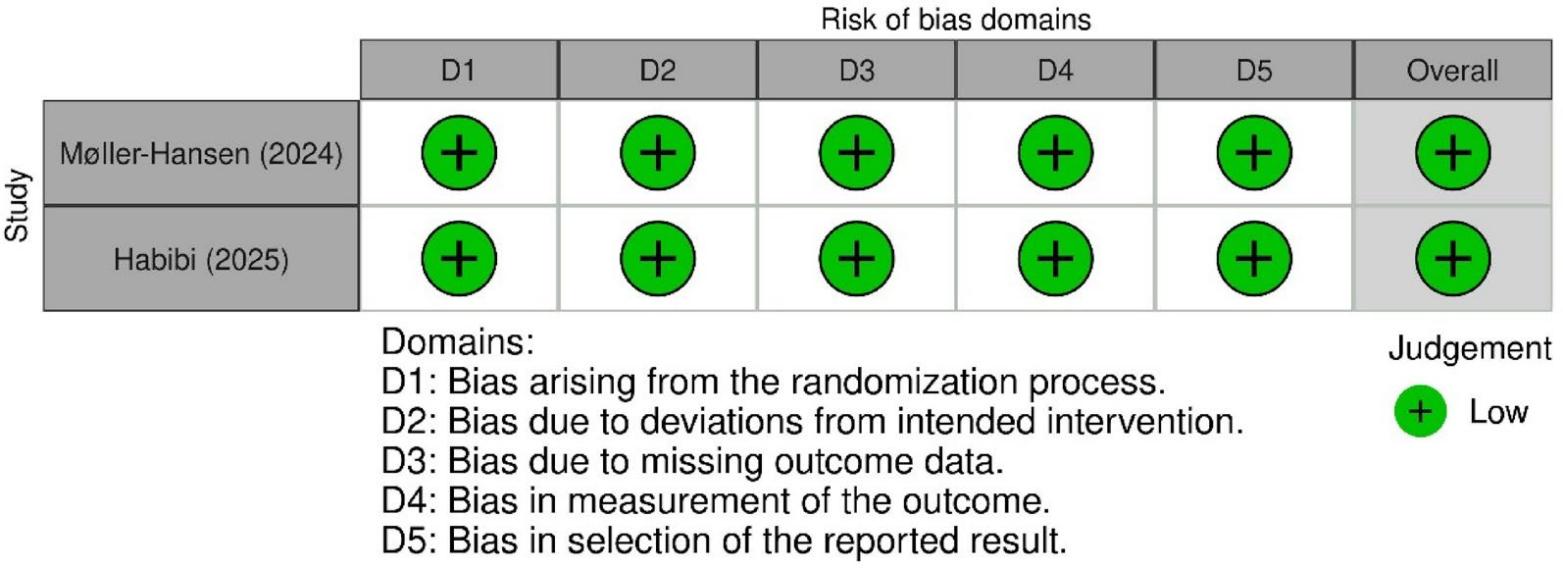
Risk of bias assessment of RCTs

**Fig. 3:**
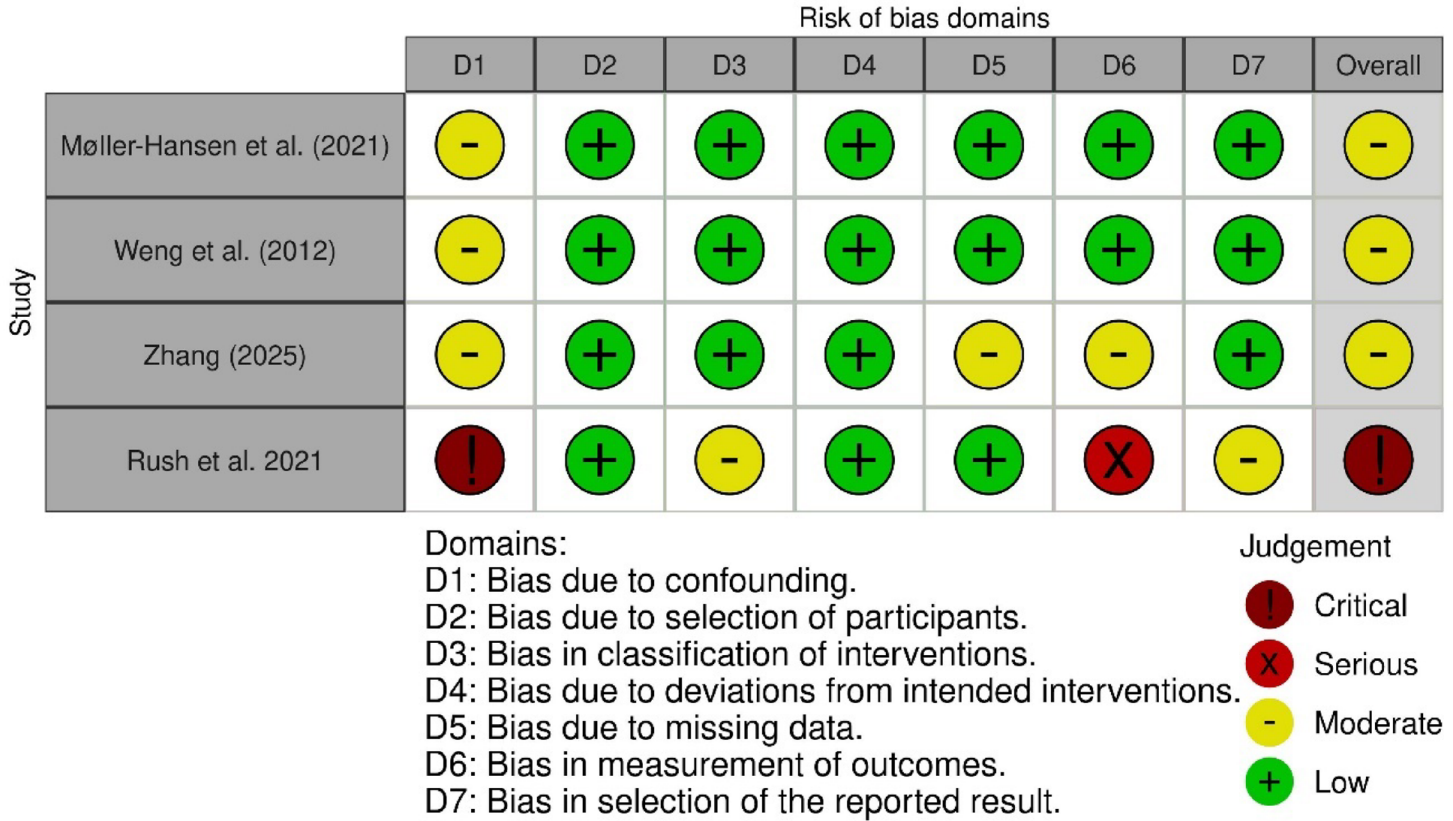
Risk of bias assessment of non-RCTs

 Both RCTs—Møller-Hansen et al. [23] and Habibi et al. [26]—were rated as low risk across all domains based on appropriate randomization procedures, allocation concealment, adherence to intervention protocols, and complete outcome reporting (Fig. 2). For non-RCTs, the ROBINS-I tool revealed moderate risk primarily in domains related to confounding, participant selection, and, in some studies, missing data and selection of reported results (Fig. 3). This reflected limited adjustment for baseline differences, insufficient clarity in eligibility criteria in studies such as Zhang [25], and incomplete reporting regarding missing outcome data. Moderate concerns in intervention classification were attributed to inadequate description or potential misclassification of treatment allocation. Additionally, blinding of outcome assessors was not consistently reported across studies. In Rush et al. [21], the study lacked a control group, resulting in critical confounding, and also demonstrated serious risk related to outcome measurement in the context of an uncontrolled design with multiple co-interventions.

### Schirmer’s test

**Fig. 4:**
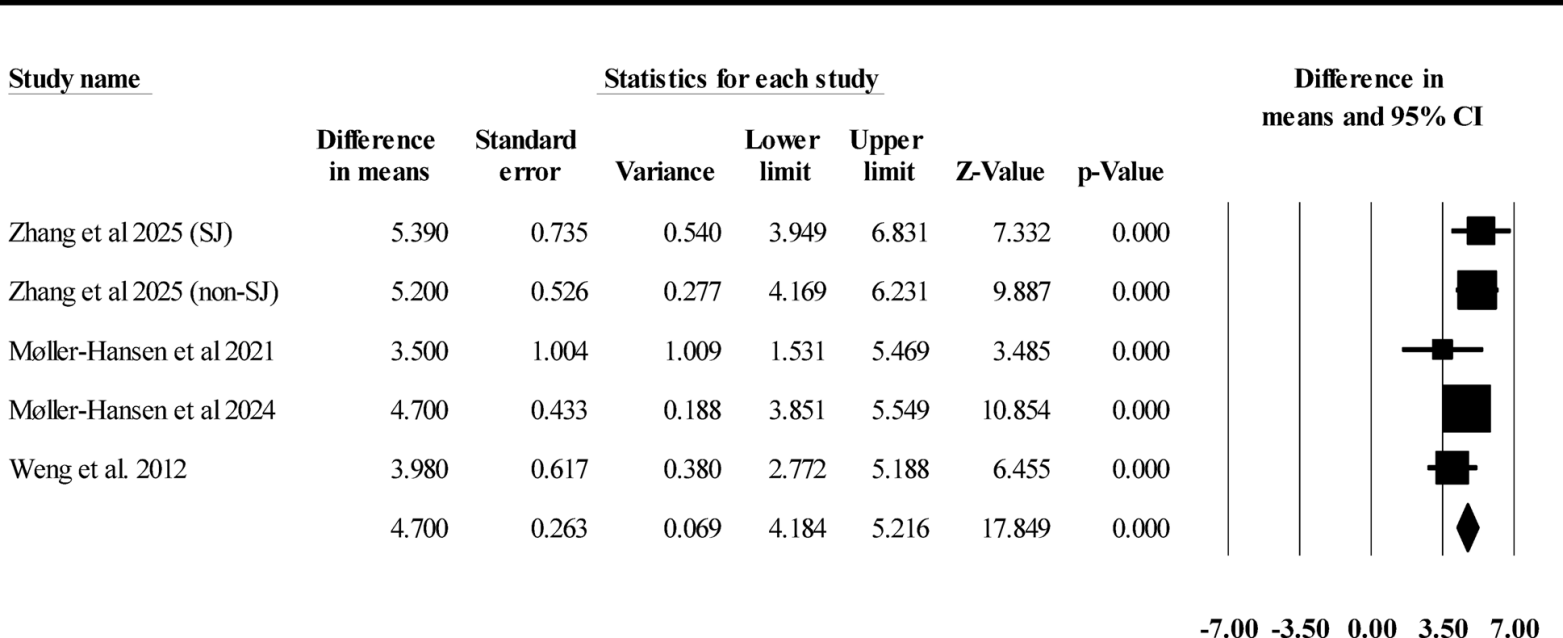
Forest plot on Schirmer’s test change post stem cell therapy. DED: Dry eye disease, MSC: Mesenchymal stem cells, TBUT: Tear break-up time, OSDI: Ocular surface disease index, CFS: Corneal fluorescein staining, STT: Schirmer tear test, NIBUT: Non-invasive tear break-up time, TMH: Tear meniscus height, ESSDAI: EULAR Sjögren’s Syndrome Disease Activity Index, ESSPRI: EULAR Sjögren’s Syndrome Patient Reported Index, ESR: Erythrocyte sedimentation rate, IL-6: Interleukin-6, MMP-9: Matrix metalloproteinase-9

Four studies involving 55 patients with DED reported the change in STT post stem cell therapy [22–25]. We pooled STT outcomes using a fixed-effect model because statistical heterogeneity was low (I² = 12.59%), and effect estimates were directionally consistent across included studies. Given the clear clinical heterogeneity in cell sources and delivery routes, we additionally examined random effects pooling as a sensitivity analysis to evaluate robustness of the pooled estimate. The meta-analysis in Fig. 4 evaluates the effect of stem cell therapy on DED using the Schirmer’s test as a primary outcome, with results pooled from each study’s final follow-up period. The overall pooled mean difference in Schirmer’s test scores before and after stem cell therapy was 4.70 mm (95% CI: 4.18–5.22), *p* < 0.001, indicating a clinically and statistically significant improvement in tear production following therapy. As expected, the heterogeneity across study outcomes was low (I² = 12.59%, *P* = 0.33, τ² = 0.054).

The duration of stem cell therapy appeared to influence the degree of improvement in Schirmer’s test scores as indicated by subgroup analysis (Supplementary Fig. S1). Subgroup analysis by follow-up intervals demonstrated that short- to medium-term treatment durations (1 to 16 weeks) consistently produced clinically significant improvements in tear production. Notably, at 4 weeks, the mean difference reached 4.99 mm, while at 16 weeks, it was 4.34 mm, both of which exceeded the generally accepted threshold for meaningful improvement in moderate to severe DED. These findings suggest that early therapeutic effects of stem cell therapy are not only measurable but also sustained over the course of a few weeks. The studies included at these timepoints had larger sample sizes and narrower CIs, indicating more reliable and precise estimates. Additionally, the heterogeneity within these subgroups remained negligible (I^2^ = 0%, *P* = 0.68, τ² = 0.0), supporting the robustness of the effect regardless of study design.

**Fig. 5:**
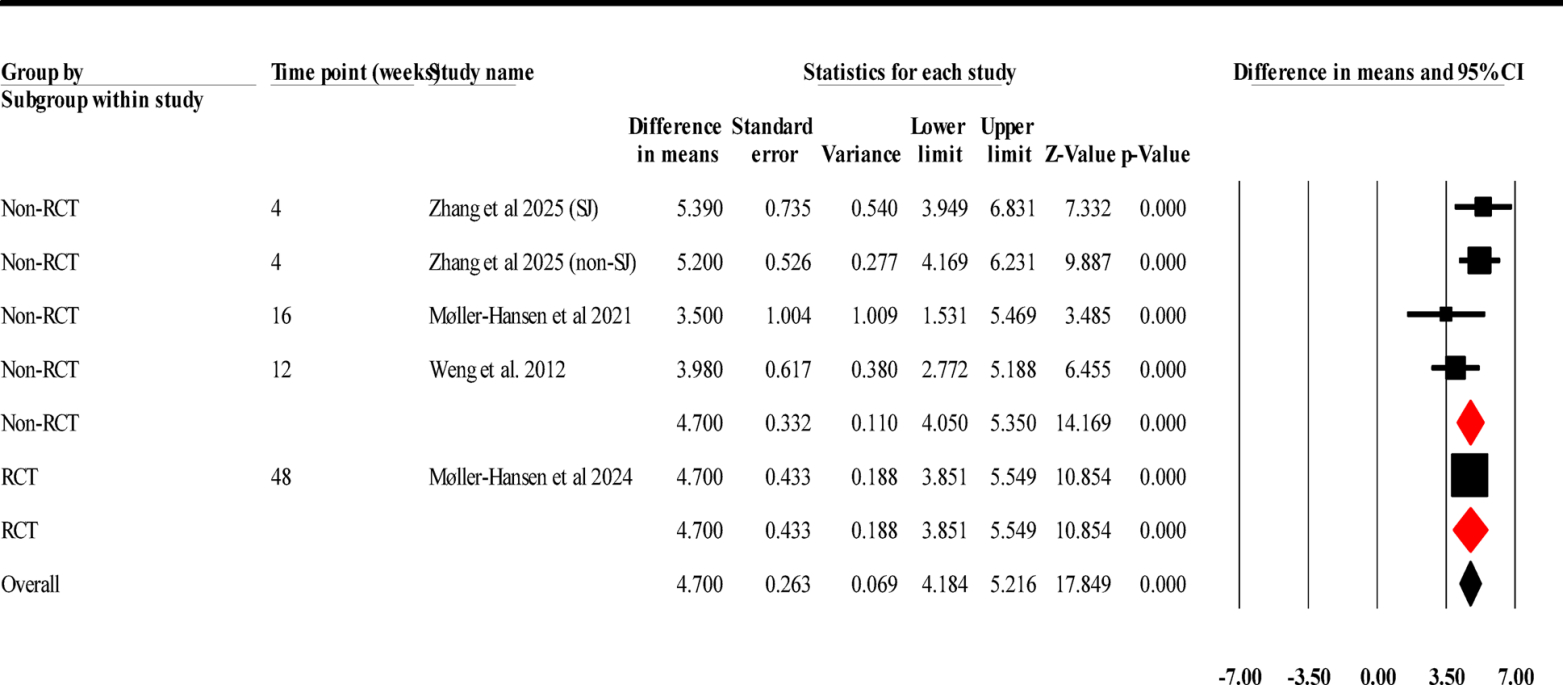
Subgroup analysis of STT by study type. DED: Dry eye disease, MSC: Mesenchymal stem cells, TBUT: Tear break-up time, OSDI: Ocular surface disease index, CFS: Corneal fluorescein staining, STT: Schirmer tear test, NIBUT: Non-invasive tear break-up time, TMH: Tear meniscus height, ESSDAI: EULAR Sjögren’s Syndrome Disease Activity Index, ESSPRI: EULAR Sjögren’s Syndrome Patient Reported Index, ESR: Erythrocyte sedimentation rate, IL-6: Interleukin-6, MMP-9: Matrix metalloproteinase-9

The subgroup analysis by study type as shown in Fig. 5 reveals important insights into the robustness of evidence supporting stem cell therapy for DED. Both randomized controlled trials (RCTs) and non-RCTs demonstrated significant improvements in STT scores. The pooled MD from non-RCTs was 4.70 mm (95% CI: 3.85–5.35), similar to the MD from RCTs at 4.70 mm (95% CI: 3.85–5.55). Moreover, the heterogeneity was low for RCTs (I² = 0%), primarily because only one study was included in this subgroup. Heterogeneity was low among non-RCTs (I^2^ = 34.40%), but higher than that observed in RCTs.

### TBUT

**Fig. 6:**
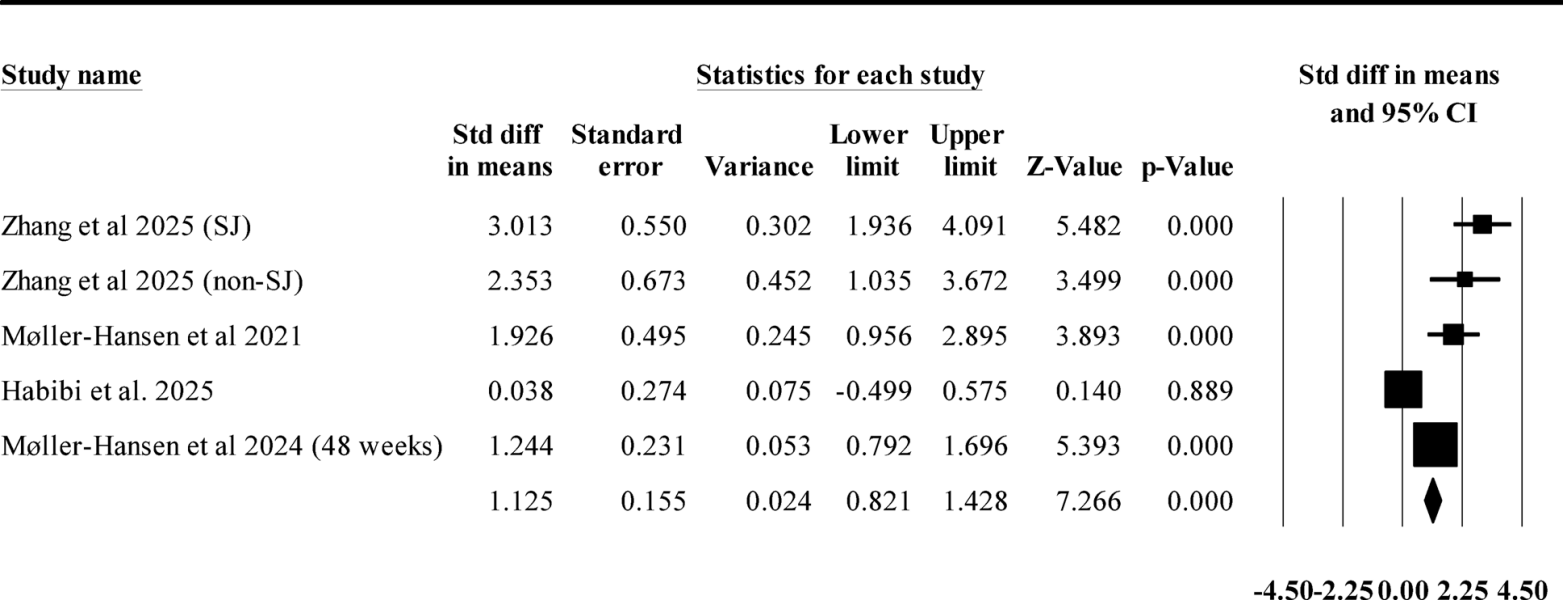
Forest plot on TBUT post stem cell therapy. DED: Dry eye disease, MSC: Mesenchymal stem cells, TBUT: Tear break-up time, OSDI: Ocular surface disease index, CFS: Corneal fluorescein staining, STT: Schirmer tear test, NIBUT: Non-invasive tear break-up time, TMH: Tear meniscus height, ESSDAI: EULAR Sjögren’s Syndrome Disease Activity Index, ESSPRI: EULAR Sjögren’s Syndrome Patient Reported Index, ESR: Erythrocyte sedimentation rate, IL-6: Interleukin-6, MMP-9: Matrix metalloproteinase-9

Four studies involving 61 patients with DED reported the effect of stem cell therapy on TBUT [22, 23, 25, 26]. Figure 6 presents the forest plot of TBUT outcomes expressed as SMDs with corresponding 95% CIs across the included clinical studies. Overall, the pooled analysis demonstrates a large and statistically significant improvement in TBUT following stem cell–based therapy, with a pooled SMD of 1.125 (95% CI: 0.821–1.428; Z = 7.266; *p* < 0.001), indicating a strong treatment effect favoring stem cell intervention.

At the individual study level, most studies demonstrated substantial standardized effect sizes with narrow CIs that did not cross the null value. Zhang et al. [25] reported substantial improvements in both Sjögren’s syndrome (SMD = 3.013; 95% CI: 1.936–4.091) and non-Sjögren’s syndrome cohorts (SMD = 2.353; 95% CI: 1.035–3.672), suggesting pronounced enhancement of tear film stability across etiological subtypes. Similarly, Møller-Hansen et al. [22] demonstrated a robust positive effect (SMD = 1.926; 95% CI: 0.956–2.895), and long-term follow-up data from Møller-Hansen et al. [23] (48 weeks) confirmed persistence of benefit over time (SMD = 1.244; 95% CI: 0.792–1.696). In contrast, Habibi et al. [26] showed a negligible and non-significant effect (SMD = 0.038; 95% CI: −0.499 to 0.575; *p* = 0.889), indicating minimal TBUT improvement in this cohort and contributing to between-study variability. Despite this, the overall pooled estimate remained strongly significant, reflecting the dominance of consistently positive effects across the majority of included studies. Collectively, these findings indicate that stem cell–based therapies are associated with large standardized improvements in tear film stability, although variability in effect magnitude across studies suggests potential influences of disease subtype, intervention modality, and follow-up duration.

Subgroup analysis based on treatment duration (1–48 weeks) demonstrated consistent improvements across all timepoints, with the most pronounced effects observed at 16 weeks (MD = 3.72 seconds) and 48 weeks (MD = 3.50 seconds) (Supplementary Fig. S2). Week 12 showed the least improvement (MD = 0.10 seconds). Clinically, these findings suggest that stem cell therapy offers a promising regenerative treatment for DED by stabilizing the tear film and slowing disease progression. The sustained improvements in TBUT across various durations further support its potential for both short- and long-term management of DED, particularly in moderate to severe cases.

**Fig. 7:**
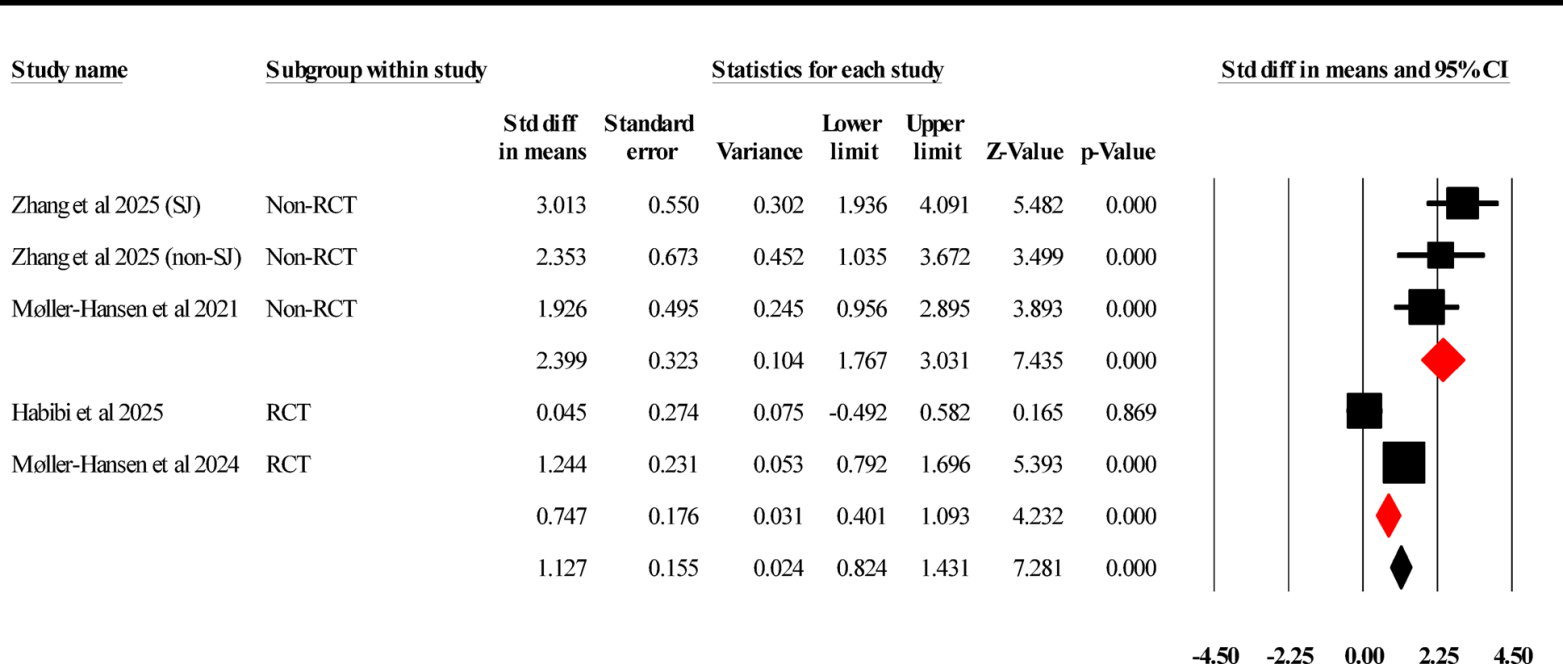
Subgroup analysis of TBUT by study type. DED: Dry eye disease, MSC: Mesenchymal stem cells, TBUT: Tear break-up time, OSDI: Ocular surface disease index, CFS: Corneal fluorescein staining, STT: Schirmer tear test, NIBUT: Non-invasive tear break-up time, TMH: Tear meniscus height, ESSDAI: EULAR Sjögren’s Syndrome Disease Activity Index, ESSPRI: EULAR Sjögren’s Syndrome Patient Reported Index, ESR: Erythrocyte sedimentation rate, IL-6: Interleukin-6, MMP-9: Matrix metalloproteinase-9

Due to observed between-study heterogeneity in the overall TBUT analysis, we performed a subgroup analysis by study type to identify potential source of heterogeneity. Figure 7 presents a subgroup meta-analysis of TBUT outcomes stratified by study design, with effects expressed as SMDs and corresponding 95% CIs. Among non-RCTs, all included studies demonstrated large and statistically significant improvements in TBUT following stem cell–based therapy. Specifically, Zhang et al. [25] reported pronounced effects in both Sjögren’s syndrome (SMD = 3.013; 95% CI: 1.936–4.091) and non-Sjögren’s syndrome cohorts (SMD = 2.353; 95% CI: 1.035–3.672), while Møller-Hansen et al. [22] also showed a robust positive effect (SMD = 1.926; 95% CI: 0.956–2.895). The pooled estimate for non-RCTs indicated a substantial overall effect (SMD = 2.399; 95% CI: 1.767–3.031; *p* < 0.001), reflecting consistent and substantial improvement in tear film stability across observational studies. In contrast, the RCT subgroup demonstrated greater variability in effect magnitude between studies. Habibi et al. [26] showed no statistically significant effect on TBUT (SMD = 0.045; 95% CI: −0.492 to 0.582; *p* = 0.869), whereas Møller-Hansen et al. [23] reported a moderate but significant improvement at 48 weeks (SMD = 1.244; 95% CI: 0.792–1.696). The pooled effect for RCTs remained statistically significant but of smaller magnitude compared with non-RCTs (SMD = 0.747; 95% CI: 0.401–1.093; *p* < 0.001).

Overall, the combined synthesis across all study designs yielded a pooled SMD of 1.127 (95% CI: 0.824–1.431; *p* < 0.001), indicating a large standardized improvement in TBUT following stem cell–based therapy. The clear discrepancy in effect size between non-RCTs and RCTs suggests that observational studies may overestimate treatment effects relative to randomized evidence, underscoring the need for additional well-designed, adequately powered RCTs to more precisely define the magnitude of TBUT improvement.

### OSDI

**Fig. 8:**
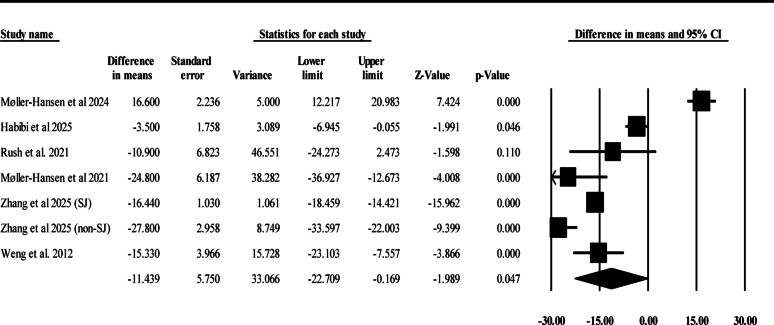
Forest plot on OSDI outcome. DED: Dry eye disease, MSC: Mesenchymal stem cells, TBUT: Tear break-up time, OSDI: Ocular surface disease index, CFS: Corneal fluorescein staining, STT: Schirmer tear test, NIBUT: Non-invasive tear break-up time, TMH: Tear meniscus height, ESSDAI: EULAR Sjögren’s Syndrome Disease Activity Index, ESSPRI: EULAR Sjögren’s Syndrome Patient Reported Index, ESR: Erythrocyte sedimentation rate, IL-6: Interleukin-6, MMP-9: Matrix metalloproteinase-9

Five studies reported the impact of stem cell therapy on reducing OSDI among 88 patients with DED [21–23, 25, 26]. Figure 8 illustrates the meta-analysis of OSDI outcomes following stem cell–based therapy, with effects expressed as MD and corresponding 95% CIs. Overall, the pooled analysis demonstrates a statistically significant reduction in OSDI scores, with a pooled MD of −11.44 (95% CI: −22.71 to − 0.17; Z = − 1.99; *p* = 0.047), indicating an overall improvement in patient-reported dry eye symptoms after treatment.

At the individual study level, most investigations reported clinically meaningful reductions in OSDI. Notably, Zhang et al. [25] showed substantial symptom improvement in both Sjögren’s syndrome (MD = −16.44; 95% CI: −18.46 to − 14.42) and non-Sjögren’s syndrome cohorts (MD = − 27.80; 95% CI: −33.60 to − 22.00). Similarly, Møller-Hansen et al. [22] and Weng et al. [24] demonstrated significant decreases in OSDI (MD = − 24.80 and − 15.33, respectively), supporting a robust symptomatic benefit across diverse etiologies.

In contrast, heterogeneity in effect direction and magnitude was evident. Møller-Hansen et al. [23] reported an increase in OSDI scores (MD = 16.60; 95% CI: 12.22 to 20.98), indicating statistically significant symptom worsening at that specific follow-up time point, while Habibi et al. [26] demonstrated a small but statistically significant reduction (MD = − 3.50; 95% CI: −6.95 to − 0.06). Rush et al. [21] showed a non-significant trend toward improvement (MD = − 10.90; 95% CI: −24.27 to 2.47; p = 0.110), reflecting imprecision likely related to small sample size and study design.

Collectively, these findings indicate that stem cell–based therapies are associated with an overall reduction in dry eye symptom burden, although the wide CIs and opposing effects observed in individual studies highlight substantial between-study heterogeneity. This variability underscores the influence of differences in study design, patient population, disease etiology, intervention type, and follow-up duration, and supports cautious interpretation of pooled symptom outcomes pending further large, standardized randomized trials.

When stratified by treatment duration, inconsistent outcomes were observed across time points (ranging from 1 to 48 weeks). Particularly, studies with short durations (e.g., 2–16weeks) demonstrated marked reductions in OSDI, suggesting an onset of symptomatic relief, while studies at 1 and 48 weeks showed an increase in OSDI, demonstrating variability in efficacy (Supplementary Fig. S3).

**Fig. 9:**
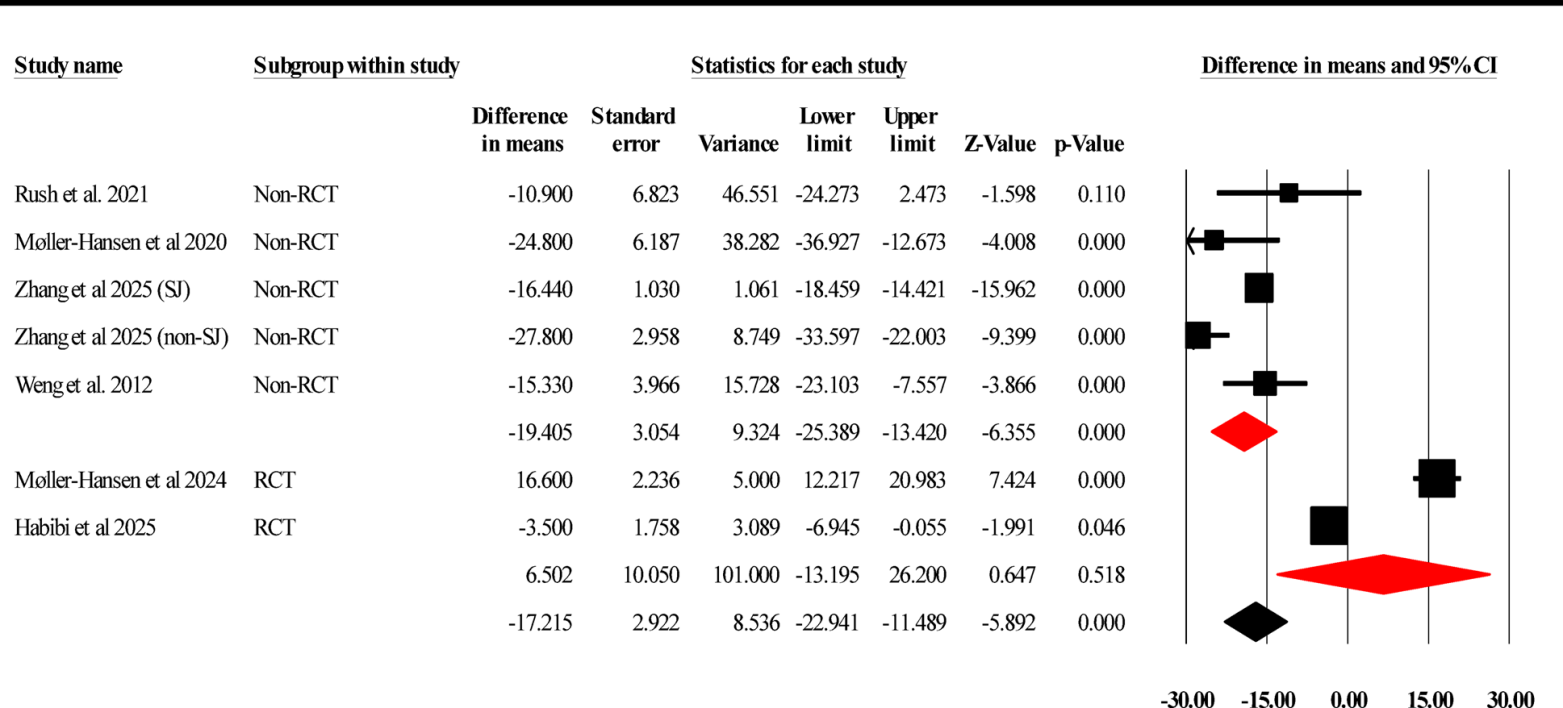
OSDI outcome sub grouped based on study design. DED: Dry eye disease, MSC: Mesenchymal stem cells, TBUT: Tear break-up time, OSDI: Ocular surface disease index, CFS: Corneal fluorescein staining, STT: Schirmer tear test, NIBUT: Non-invasive tear break-up time, TMH: Tear meniscus height, ESSDAI: EULAR Sjögren’s Syndrome Disease Activity Index, ESSPRI: EULAR Sjögren’s Syndrome Patient Reported Index, ESR: Erythrocyte sedimentation rate, IL-6: Interleukin-6, MMP-9: Matrix metalloproteinase-9

Figure 9 illustrates the meta-analysis of OSDI outcomes following stem cell–based therapy, with effects expressed as MDs and corresponding 95% CIs. Overall, the pooled analysis demonstrates a statistically significant reduction in OSDI scores, with a combined MD of −11.44 (95% CI: −22.71 to − 0.17; Z = − 1.99; *p* = 0.047), indicating an overall improvement in patient-reported dry eye symptoms after treatment.

At the individual study level, most investigations reported clinically meaningful reductions in OSDI. Notably, Zhang et al. [25] showed substantial symptom improvement in both Sjögren’s syndrome (MD = − 16.44; 95% CI: −18.46 to − 14.42) and non-Sjögren’s syndrome cohorts (MD = − 27.80; 95% CI: −33.60 to − 22.00). Similarly, Møller-Hansen et al. [23] and Weng et al. [24] demonstrated significant decreases in OSDI (MD = − 24.80 and − 15.33, respectively), supporting a robust symptomatic benefit across diverse etiologies.

In contrast, heterogeneity in effect direction and magnitude was evident. Møller-Hansen et al. [23] reported an increase in OSDI scores (MD = 16.60; 95% CI: 12.22 to 20.98), suggesting symptom worsening at that specific follow-up, while Habibi et al. [26] demonstrated a small but statistically significant reduction (MD = − 3.50; 95% CI: −6.95 to − 0.06). Rush et al. [21] showed a non-significant trend toward improvement (MD = − 10.90; 95% CI: −24.27 to 2.47), reflecting imprecision likely related to small sample size and study design.

Collectively, these findings indicate that stem cell–based therapies are associated with an overall reduction in dry eye symptom burden, however, the RCT subgroup showed a non-significant pooled increase in OSDI, and the wide CIs and opposing effects observed in individual studies highlight substantial between-study heterogeneity. This variability underscores the influence of differences in study design, patient population, disease etiology, intervention type, and follow-up duration, and supports cautious interpretation of pooled symptom outcomes pending further large, standardized randomized trials.

### Corneal fluorescein staining (CFS)

**Fig. 10:**
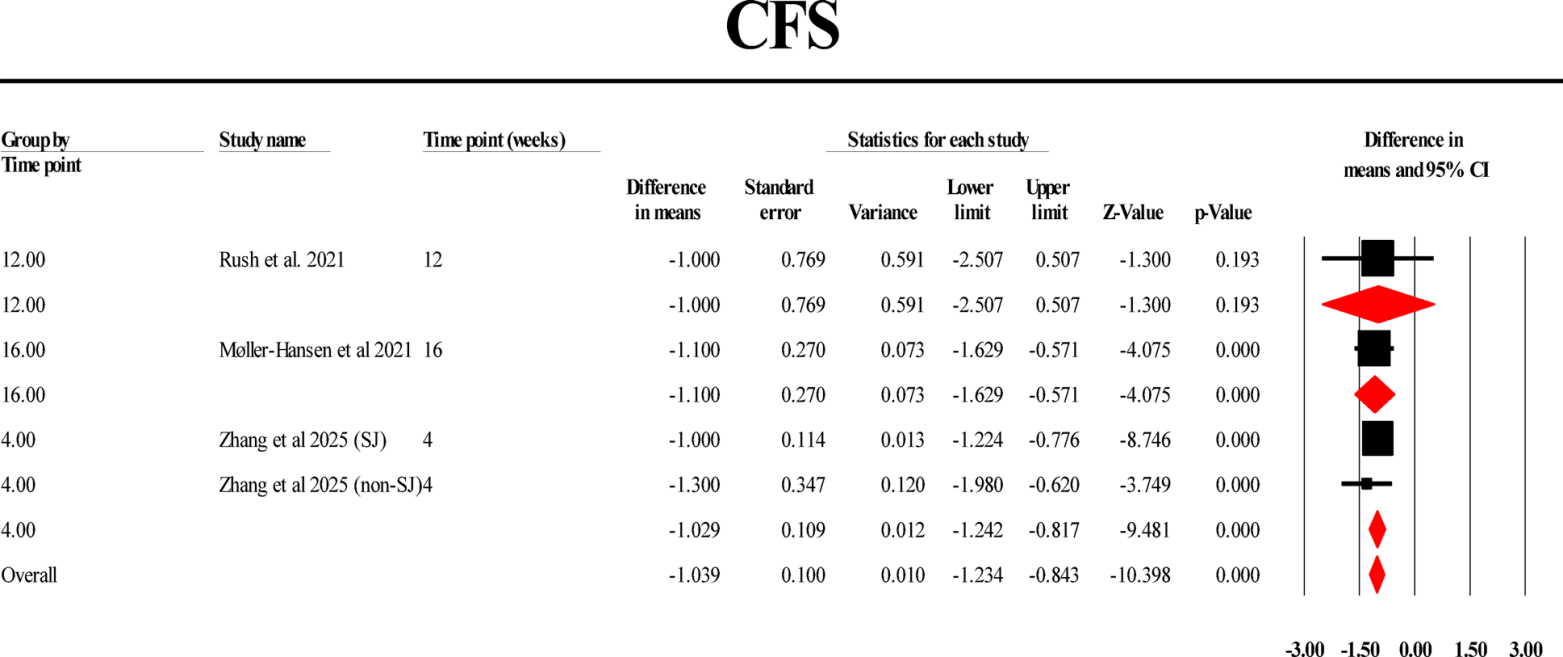
Forest plot on CFS outcome. DED: Dry eye disease, MSC: Mesenchymal stem cells, TBUT: Tear break-up time, OSDI: Ocular surface disease index, CFS: Corneal fluorescein staining, STT: Schirmer tear test, NIBUT: Non-invasive tear break-up time, TMH: Tear meniscus height, ESSDAI: EULAR Sjögren’s Syndrome Disease Activity Index, ESSPRI: EULAR Sjögren’s Syndrome Patient Reported Index, ESR: Erythrocyte sedimentation rate, IL-6: Interleukin-6, MMP-9: Matrix metalloproteinase-9

Three studies reported the effect of stem cell therapy on reducing CFS among 40 patients with DED [21, 22, 25]. The pooled analysis, using a fixed-effect model, of CFS pre- and post-treatment with stem cell therapy for DED revealed a statistically significant MD of −1.04 (95% CI: −1.23 to −0.84; *p* < 0.001) (Fig. 10). The pooled estimate represents the change from baseline to the follow-up after treatment. This negative value indicates a reduction in CFS scores, suggesting improvement in ocular surface integrity following stem cell intervention. Clinically, a reduction of approximately 1.04 units in CFS may be clinically meaningful, as decreases in CFS scores are generally associated with symptomatic relief and epithelial recovery in DED. The overall analysis reported negligible between-study heterogeneity (I² = 0%, *P*τ² = 0.0, p = 0.87), indicating no significant heterogeneity among the included studies. This suggests that the effect of stem cell therapy on CFS was consistent across the included studies, strengthening the reliability of the pooled estimate.

### Publication bias

Due to the limited number of included studies (<10 per outcome), formal statistical tests for publication bias were not considered reliable. Therefore, potential small-study effects were interpreted qualitatively.

## Discussion

Stem cell therapies may improve both objective and subjective outcomes in DED; however, these findings should be interpreted cautiously due to small sample sizes, heterogeneity in study design, and variability in intervention protocols. Across all included studies, no treatment-related adverse effects or serious complications were reported, highlighting the favorable safety profile of stem cell-based therapies. Whether administered topically, periocularly, intraglandularly, intravenously, or systemically, MSC therapies were well-tolerated even with extended follow-up durations of up to nine months [22, 24, 27–34]. Across the included studies, topical ocular administration was the most commonly utilized route.

This meta-analysis provides clinical evidence supporting the use of stem cell therapy as a potentially effective treatment for DED, particularly in moderate to severe and refractory cases. Significant improvements were observed across STT, TBUT, and OSDI. Tear production, as measured by the STT, improved by nearly 4.70 mm, while TBUT a large standardized effect size (SMD), rather than a direct time-based increase, both reflecting restored tear function and ocular surface stability. Symptomatically, patients experienced a substantial 11.44-point reduction in OSDI scores, indicating rapid and durable relief. Improvements in corneal staining further support the regenerative potential of stem cell therapy in promoting epithelial healing. These outcomes were consistent across multiple timepoints and study designs, with low heterogeneity enhancing the reliability of the results. While RCTs yielded slightly smaller effects and higher-quality evidence, non-RCTs corroborated these findings, enhancing generalizability. Importantly, publication bias assessments revealed minimal risk, affirming the robustness of the evidence base.

Clinically, these findings suggest that stem cell therapies may potentially offer a safe, biologically restorative alternative or adjunct to traditional treatments, particularly for patients unresponsive to conventional therapies. They underscore the need for further large-scale, long-term RCTs to standardize treatment protocols and maximize therapeutic outcomes. While our findings suggest significant improvements in objective and subjective measures of DED, these results must be interpreted cautiously. The included studies varied substantially in terms of stem cell source (e.g., bone marrow, adipose, umbilical cord), delivery method (topical, periocular, systemic), dosage, and frequency. Such variability introduces clinical and biological heterogeneity that may affect treatment efficacy and safety profiles. Although our meta-analysis provides useful aggregate evidence, these differences limit the extent to which findings can be generalized or used to inform specific clinical protocols. Future studies should standardize cell source, dose, and delivery methods to enable more definitive recommendations.

Regarding efficacy, substantial improvements were observed in both subjective symptoms and objective clinical indicators of dry eye. Improvements in tear production were a consistent finding across most studies, as assessed by STT or equivalent measures. In clinical trials involving human patients with aqueous-deficient DED or graft-versus-host disease (GVHD)-related dry eye, significant increases in tear production and reduction in OSDI scores were observed post-treatment [22, 24]. Preclinical evidence from animal models of immune-mediated DED such as Sjögren’s syndrome and KCS suggests that MSC-based interventions enhance aqueous tear secretion [28, 30–33]. These effects were sustained over several weeks to months, with some studies demonstrating therapeutic persistence up to six or even nine months after a single MSC administration [30, 31].

Tear film stability, commonly measured via TBUT, also improved significantly in both preclinical and clinical contexts. Enhanced TBUT was observed in MSC-treated animals and humans, indicating improved mucin and lipid layer integrity likely due to goblet cell preservation and meibomian gland restoration [27, 29, 32].

To ensure clinical relevance and avoid bias from interspecies variability, only human clinical data were included in the meta-analysis. This methodological separation preserves the validity of the effect estimates. However, a key limitation of this review lies in the heterogeneity of stem cell interventions across studies, including differences in cell source, dosage, delivery methods, and treatment frequency. These inconsistencies may have influenced outcomes and limited comparability across trials. Although subgroup analyses were attempted, further stratification was hindered by small sample sizes and inconsistent reporting. This highlights the need for standardized protocols in future clinical trials. Additionally, while MSC-derived exosomes show therapeutic promise through their regenerative and anti-inflammatory properties, their clinical adoption is challenged by complexities in large-scale production, quality control, and regulatory pathways. Though current therapies are effective for many DED patients, those with chronic or refractory disease may benefit from cell-based approaches. This study aims to consolidate early clinical and preclinical evidence, serving as a foundation for future research rather than replacing existing treatment strategies. The clinical utility of MSC therapy should be viewed within the broader treatment landscape of DED. While many patients benefit from established pharmacological and procedural interventions, MSCs may serve as an adjunct or salvage therapy in cases of refractory, progressive, or severe DED, especially where epithelial healing is impaired or fibrosis is present. However, their application is constrained by complexities in production, scalability, cost, and stringent regulatory requirements, which currently limit routine clinical use. Furthermore, robust evidence identifying which DED subtypes or biomarkers predict response to MSC therapy is lacking and warrants further investigation. Until such parameters are defined, MSCs should be considered experimental, with future trials aimed at clarifying their optimal clinical niche.

Systemic administration of MSCs in GVHD-associated DED is logical given their broad and sustained immunosuppressive effects. In such cases, ocular improvements often reflect systemic immune modulation rather than localized action. In contrast, local MSC delivery—via topical, intraglandular, or subconjunctival routes—acts directly on the ocular surface, promoting epithelial repair, tear film restoration, and localized immunoregulation through paracrine signaling [35]. While systemic MSCs may provide prolonged systemic immune relief, local delivery offers a more targeted, lower-risk strategy, potentially requiring repeated dosing for sustained effects. However, head-to-head comparisons are lacking, and therapeutic choice should depend on DED subtype and underlying etiology. Local therapy may be more appropriate for isolated, non-systemic DED, whereas systemic MSCs are justified in severe, immune-mediated conditions like GVHD.

Although MSC-derived exosomes require sophisticated processing, this is due to the technology’s development stage rather than a lack of therapeutic relevance [36]. Current dry eye treatments largely provide symptom alleviation and are frequently insufficient for people with severe, refractory, or autoimmune-related disease. In contrast, stem cell-derived therapies work through regenerative and immunomodulatory pathways to tackle the underlying causes of ocular surface injury [20]. The current study is clinically important since it consolidates early findings, highlights preparation-related issues, and outlines the standardization and manufacturing procedures required to support greater clinical translation.

Generally, the results from this review underscore the transformative potential of stem cell and stem-cell-derived therapies to redefine the treatment landscape for DED. Nevertheless, realizing this potential will necessitate rigorous standardization of cell sources, delivery strategies, and characterization protocols to enhance reproducible outcomes. The standardization will also hasten translation into routine clinical practice.

This systematic review and meta-analysis has several limitations that should be acknowledged. Firstly, there was substantial heterogeneity in study designs, populations, stem cell sources, administration routes, and outcome measures, which complicates direct comparison and limits the generalizability of the findings. The limited number of high-quality human trials restricted the applicability of results to clinical practice. Small sample sizes in several studies reduce statistical power and increase the risk of bias. Inconsistencies in how outcomes such as STT, TBUT, and OSDI scores were measured and reported further challenge the synthesis of findings. Short follow-up durations in some studies limit conclusions on the long-term efficacy and safety of MSC therapy. Although the funnel plot did not suggest significant publication bias, the relatively small number of studies included may reduce the sensitivity of this assessment. Additionally, histological and immunological outcomes were not uniformly reported, limiting insight into the underlying mechanisms of action. Because of the limited number of human trials, subgroup analyses based on DED subtype, severity, or diagnostic protocol were not statistically feasible. Consequently, heterogeneity should be interpreted as an inherent limitation of the current evidence base rather than a methodological omission. Finally, the exclusion of non-English and unpublished studies introduces potential selection bias and may have led to the omission of relevant data.

## Conclusion

This systematic review and meta-analysis demonstrate that MSC therapy is associated with significant improvements in clinical outcomes among patients with DED. No serious treatment-related adverse events were reported, but standardized adverse event definitions and longer follow-up are needed to better characterize safety. However, heterogeneity in stem cell type, delivery method, diagnostic criteria, and follow-up limits generalizability. While early results are promising, stem cell therapy for DED remains experimental. Larger, standardized clinical trials are required to confirm efficacy, optimize protocols, and support regulatory and clinical integration.

## Supplementary Information

Supplementary material 1

Supplementary material 2

## Data Availability

All data generated or analyzed in this study are fully contained within the main manuscript and its tables and figures. Supplementary materials are provided and include Supplementary Tables and Figures referenced in the manuscript for this systematic review and meta-analysis; therefore, no additional datasets are available.
