# Supplementary material for: Efficacy and safety of stem cell therapy for dry eye disease: a systematic review and meta-analysis
Source: Stem Cell Res Ther. 2026 May 1;17:224. doi: 10.1186/s13287-026-04915-8 (PMC13281483; doi:10.1186/s13287-026-04915-8)
Supplement: Supplementary file 2 — Supplementary material 2 [file 13287_2026_4915_MOESM2_ESM.docx]

Supplementary Table 1. Search Strategies Employed Across Databases.

| **Database** | **Search Terms** |
| --- | --- |
| **PubMed** | ("dry eye"[All Fields] OR "keratoconjunctivitis sicca"[All Fields] OR "aqueous-deficient dry eye"[All Fields] OR "DED"[All Fields]) AND ("stem cell"[All Fields] OR "mesenchymal stem cells"[All Fields] OR "MSC"[All Fields] OR "adipose-derived stem cells"[All Fields] OR "exosomes"[All Fields] OR "regenerative therapy"[All Fields]) |
| **Google Scholar** | “dry eye disease” OR “keratoconjunctivitis sicca” OR “DED” AND “stem cell therapy” OR “mesenchymal stem cells” OR “MSC” OR “regenerative therapy” OR “exosomes” |
| **Cochrane Library** | (dry eye disease OR keratoconjunctivitis sicca OR aqueous-deficient dry eye OR DED):ti,ab,kw AND (“stem cell therapy” OR “mesenchymal stem cells” OR MSC OR “adipose-derived stem cells” OR exosomes OR “regenerative therapy”) |
| **SCOPUS** | ( TITLE-ABS-KEY ( "dry eye disease" OR "DED" OR "keratoconjunctivitis sicca" OR "aqueous-deficient dry eye" ) AND TITLE-ABS-KEY ( "stem cell therapy" OR "stem cells" OR "mesenchymal stem cells" OR "MSC" OR "adipose-derived stem cells" OR "exosomes" OR "regenerative therapy" ) ) |
| **EMBASE** | ('dry eye'/exp OR 'dry eye' OR 'dry eye disease' OR 'keratoconjunctivitis sicca' OR 'aqueous-deficient dry eye' OR 'DED')  AND  ('stem cell transplantation'/exp OR 'stem cell therapy' OR 'stem cells' OR 'mesenchymal stem cell'/exp OR 'MSC' OR 'adipose derived stem cell' OR 'exosome'/exp OR 'exosomes' OR 'regenerative therapy') |
| **Web of Science** | ("dry eye disease" OR "DED" OR "keratoconjunctivitis sicca" OR "aqueous-deficient dry eye") AND ("stem cell therapy" OR "stem cells" OR "mesenchymal stem cells" OR "MSC" OR "adipose-derived stem cells" OR "exosomes" OR "regenerative therapy") |

**Figure S1.** Forest plot showing subgroup analysis by follow-up period for changes in Schirmer’s test following stem cell therapy.

**Figure S2.** Subgroup analysis of tear breakup time (TBUT) according to follow-up duration.

**Figure S3.** Subgrouped analysis of Ocular Surface Disease Index (OSDI) outcomes based on follow-up period.

**Figure S4.** Funnel plot assessing publication bias for the Schirmer’s tear test outcome.

**Figure S5.** Funnel plot assessing publication bias for the tear breakup time (TBUT) outcome.

**Figure S6.** Funnel plot assessing publication bias for the Ocular Surface Disease Index (OSDI) outcome.

**Figure S7.** Funnel plot assessing publication bias for the corneal fluorescein staining outcome.
